# Chronic disorders of consciousness: a case report with longitudinal evaluation of disease progression using 7 T magnetic resonance imaging

**DOI:** 10.1186/s12883-020-01973-0

**Published:** 2020-10-29

**Authors:** Xiaoxia Li, Xufei Tan, Pinyi Wang, Xiaohua Hu, Yan Dong, Xiaotong Zhang, Benyan Luo

**Affiliations:** 1 Department of Neurology and Brain Medical Centre, The First Affiliated Hospital, School of Medicine, Zhejiang University, Qingchun Road, Hangzhou, 310003 China; 2 Department of Clinical Medicine, Zhejiang University City College School of Medicine, Hangzhou, China; 3 Interdisciplinary Institute of Neuroscience and Technology, Key Laboratory for Biomedical Engineering of Ministry of Education, Zhejiang University, Hangzhou, China; 4 Department of Rehabilitation, Hangzhou Hospital of Zhejiang CAPR, Hangzhou, China; 5 School of Medicine, Zhejiang University, Hangzhou, China

**Keywords:** Arcuate fasciculus, Connectome, Diffusion, Disorders of consciousness, Severe brain injury, Superior longitudinal fasciculus, Traumatic brain injury

## Abstract

**Background:**

Outcome prediction for patients with disorders of consciousness (DOC) is essential yet challenging. Evidence showed that patients with DOC lasting 1 year or longer after a brain injury were less likely to recover. However, the reasons why outcomes of DOC patients differ greatly remain unclear. With a variety of analytical methods and through quantitative behavioral assessments, we aimed to track the progression of a patient with severe brain injury, in order to advance our understanding of the underlying mechanisms of DOC.

**Case presentation:**

We performed a longitudinal study for a 52-year-old male DOC patient who has remained in the state for 1.5 years with comprehensive rehabilitative therapies. The patient underwent 3 times of assessments of Coma Recovery Scale-Revised (CRS-R) and ultra-high-field 7 T magnetic resonance imaging (MRI). Both topologic properties and brain microstructure were analyzed to track disease progression. We observed dynamic increases of fiber densities with measurements at three time points (t1:1.5 M, t2:7.5 M t3:17.5 M). Specifically, fiber densities of the superior longitudinal fasciculus and arcuate fasciculus nerve fiber bundles improved mostly in the visual, verbal, and auditory subscales, which was consistent with the CRS-R scores. Moreover, the graph-theory analyses demonstrated that network topologic properties showed an improvement although the disease duration exceeded 1 year.

**Conclusions:**

DOC patients with a course longer than 1 year remain possible to improve, and including evaluation methods such as WM connectome analysis and graph theory could be potentially valuable for a more precise assessment of patients with a longer course of DOC.

## Background

Disorders of consciousness (DOC) are a global public health concern and have profound influences on modern society from physical, psychosocial, and socioeconomic perspectives [1]. Researchers suggested that the recovery of brain function in DOC patients after severe brain injury could only develop within a specific time frame, which typically does not exceed 1 year [2]. If physicians have incorrectly assessed the DOC progress, patients could be directly transferred from high-intensity first aid facilities to poorly managed care settings, making it impossible to provide necessary professional assessments and treatments [3], and thus is unfavorable for prognosis. Hence, proper assessments of DOC progression are essential, which, however, rely on the Coma Recovery Scale-Revised (CRS-R) score. The CRS-R score, in the meantime, has been widely used clinically, but may not be optimal as it is a clinician-dependent assessment and is susceptible to subjectivity. Even when combined with commonly used magnetic resonance imaging (MRI) and diffusion metrics, similar subjectivity remains. Therefore, it is necessary to use multiple methods to evaluate disease progression in a comprehensive way over DOC patients.

Observing changes in brain microstructure is essential to advance our understanding of the underlying mechanisms of DOC. Connectometry is a new method based on deterministic fiber tracking algorithm, which accurately reflects the structure and density of the white matter bundle, while considering the cross-fiber and partial volume effect [4, 5]. We aim to apply connectometry to observe changes in white matter tracts at different time points.

Besides, graph theory analysis based on tractography allows us to characterize the structural connection patterns of the human brain in vivo, to delineate the whole brain as a large-scale network consisting of nodes and edges, as well as to focus on revealing topology abnormalities [6]. Graph theory analyses provide a new perspective for the further understanding of disease progression in DOC patients.

Tan et al. described changes in brain microstructure in DOC patients from spontaneously recovered unresponsive wakefulness syndrome (UWS) to a minimally conscious state (MCS) (Tan et al., 2017); however, a 5-month evaluation period might be insufficient for DOC patients. We have conducted a long-term follow-up of 17.5 months and evaluated the disease progression of DOC patients by assessing changes in brain microstructure and brain topologic network properties, alongside with CRS-R scale applied as well.

## Case presentation

A 52-year-old male patient was enrolled with a 17.5-month history of DOC. He was a carpenter without a prior history of mental or neurologic disease. However, 1.5 months prior to enrollment, he was in coma after suffering a motor vehicle accident. The patient was immediately delivered to a local hospital, and a computed tomography scan revealed several injuries, including a small subdural hematoma in the right hemisphere, subarachnoid hemorrhage, a right temporal lobe contusion, and multiple fractures throughout the body. After a series of intensive care measures, ventilator-assisted ventilation, drug nutrition, awakening, anti-infection, nutritional support, and other symptom-corresponding treatments, the patient continued to have impaired consciousness. To allow for more appropriately targeted rehabilitation therapies, the patient was then transferred to the Department of Rehabilitation. The first 7 T MRI scan and CRS-R score were assessed 1.5 months post-accident. Notably, 7.5 months after the initial trauma, the patient’s CRS-R and MRI assessments were re-evaluated and demonstrated a significantly improved CSR-R score following a series of symptom-corresponded curing and rehabilitation exercises. Based on the relatively stable condition and considering a high cost of treatment, the guardians of the patient decided to continue rehabilitation at home. Finally, 17.5 months after brain injury, the third CRS-R evaluation and MRI scan were conducted.

### Image acquisition and processing

All of the MRI scans were performed on a 7 T MRI scanner (Siemens Healthcare, Erlangen, Germany) equipped with a Nova 1Tx/32Rx head coil (Nova Medical, USA) and an SC72 body gradient with a gradient strength and slew rate of 70 mT/m and 200 T/m/s, respectively. The imaging protocols included 3D T_1_-weighted imaging (T_1_WI), and DTI. T_1_WI was obtained with a magnetization-prepared rapid gradient echo (MPRAGE) sequence and the parameters were as follow: 0.75 mm isotropic, 208 slices, echo time (TE)/repetition time (TR) = 2.50/2590 ms, flip angle = 7°, generalized auto-calibrating partially parallel acquisitions (GRAPPA) = 2, acquisition time (TA) = 5′37″. DTI was performed with a single-shot echo-planar imaging sequence using the scanning parameters: isotropic resolution was 1.25 mm, phase encoding direction: A-P and P-A, 112 slices for each direction TE/ TR = 66.2/5100 ms, flip angle = 90°, GRAPPA = 3, TA = 6′50″ Images were acquired with b values of 0 and 2000 s/mm^2^in 60 directions.

The distortion corrections (i.e., susceptibility, eddy currents, and motion) were performed using ‘eddy’ and ‘topup’ commands implemented in FSL (version 5.0; Analysis Group, FMRIB, Oxford, UK) [7]. Then, the Diffusion Toolkit/TrackVis (Wang & Weeden, Boston, MA, USA) was used for fiber tracking reconstruction, display and analysis of diffusion data [8, 9]. The average apparent diffusion coefficient (ADC), average fractional anisotropy (FA), average radial diffusivity (RD) and axial diffusivity (AD) of the whole brain were calculated. DTI parameters were used to reveal microstructural changes in the brain, particularly in FA and ADC [10, 11]. FA was the ratio of the anisotropic component of the diffusion tensor to the total diffusion tensor and ADC reflecting the diffusion rate and range of water molecules in different directions of the DTI scan. Once the fiber was obstructed, or the myelin sheath was destroyed, both values would change [12].

We conducted white matter connectometry analysis with DSI Studio software (http://dsi-studio.labsolver.org) [4, 13]. The diffusion data were reconstructed in the Montreal Neurologic Institute space using q-space diffeomorphic reconstruction with a diffusion sampling length ratio of 1.25 and an output resolution of 2 mm [14]. A deterministic fiber tracking algorithm [15] was used to connect local fiber directions with a percentage reduction greater than 5, 10, 15, 20, 25, and 30%. Additionally, a percentage increase greater than 5, 10, and 15% with a connected length greater than 30 mm were included. We further calculated the FA, ADC, AD and RD values of the fibers with the largest proportion of alterations to evaluate the changes in microstructure.

In addition to the connectometry analysis, we further investigated the changes of topologic properties. A widely used anatomical atlas (the Automated Anatomical Labeling atlas) was adopted to parcellate the brain into 116 regions [16, 17]. Tracts were computed by seeding each voxel with an angular threshold of 45°, step size of 0.5 mm, anisotropy threshold of 0.05, and a total of 100,000 seeds placed. We used the DSI Studio software to compute graph theoretical network measures [18]. The white matter network was represented with an asymmetric 116 × 116 connectivity matrix, and each element of the connectivity matrix was populated with the number of streamlines between the corresponding pair of regions. Furthermore, the number of streamlines were also regarded as the connectivity strength. The related global network metrics were defined and included the clustering coefficient, network characteristic path length, small world-ness, global efficiency, and local efficiency. The definition and characterization of network metrics adopted in the present study are listed in Table S1.

### Macrostructural changes

**Fig. 1:**
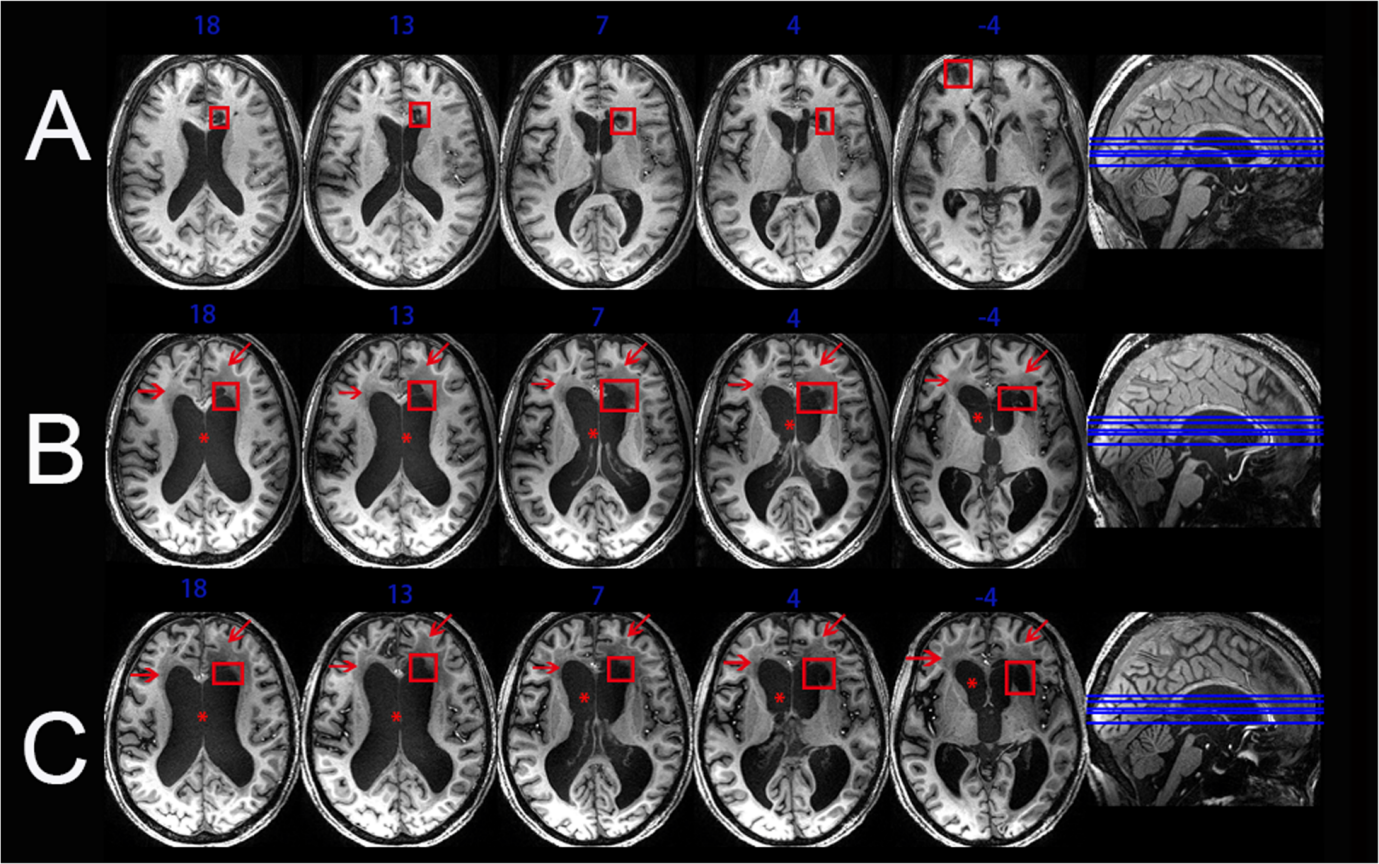
Magnetic resonance imaging (MRI) depicting macroscopic structural changes in the brain. The normalized T1-weighted axial sections of an entire MRI image obtained at (**a**) 1.5 months (**b**) 7.5 months and (**c**) 17.5 months after initial injury. Red boxes indicate the focal malacia; Red arrows indicate the oedema and/or gliosis area; Red * indicate the enlarged bilateral ventricles

Figure 1 illustrated the obvious lesions area at three time points. There are obvious focal malacia (low-T1WI signal, red box) in the right frontal lobe and left frontal horn of lateral ventricle at 1.5 months after onset. Focal malacia in the same areas was also observed in the second and third scans, which were performed at 7.5 and 17.5 months after brain injury. In addition, oedema and/or gliosis near the frontal horn of the bilateral ventricles (low-T1WI signal, red arrow) and enlarged bilateral ventricles (red *) were shown on the latter two scans.

### Quantitative behavioral evaluation of the patient

**Fig. 2:**
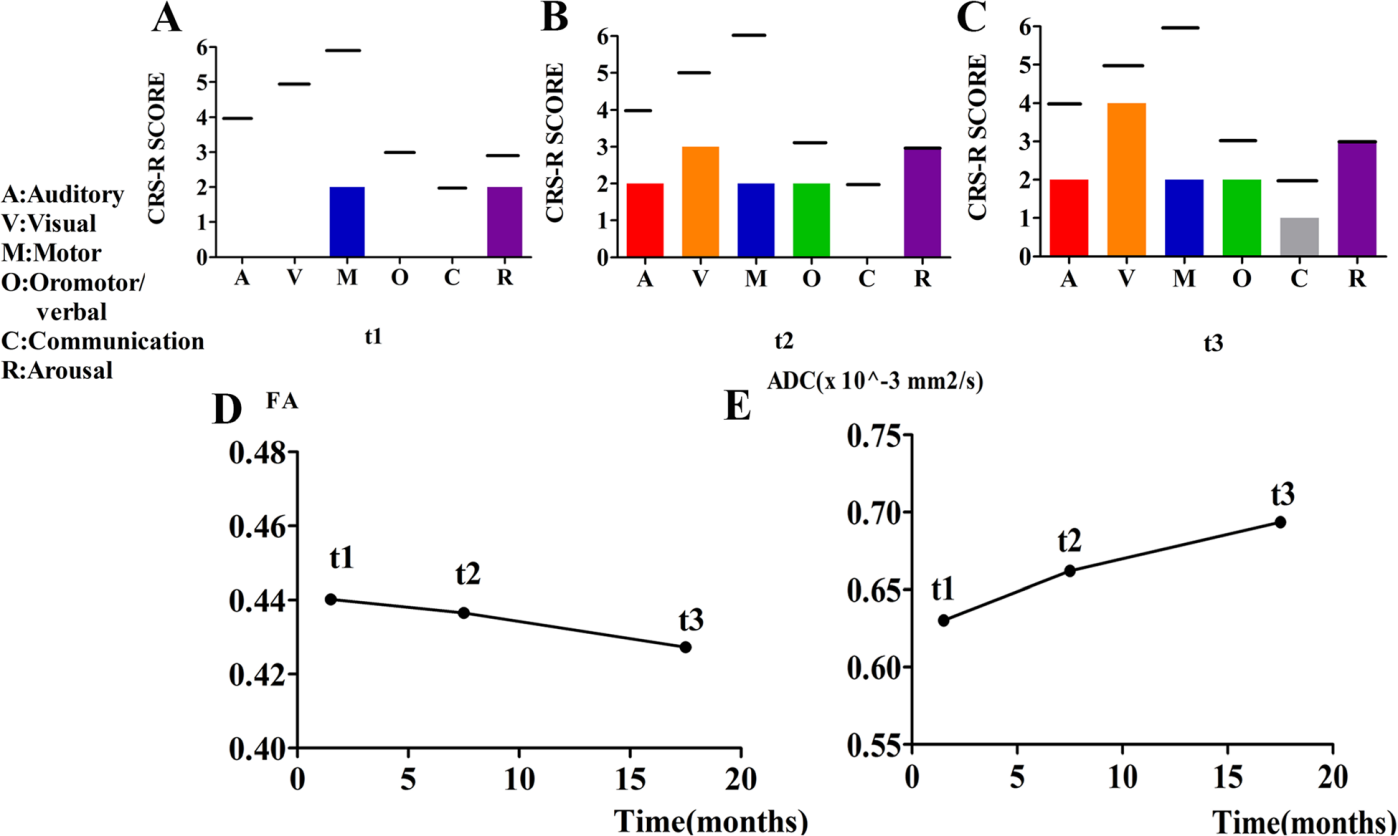
Quantitative behavioral assessments (**a**-**c**) and diffusion evaluation parameter values (**d**-**e**) obtained at different time points. A, the auditory function scale; V, the visual function scale; M, the motor function scale; O, the oromotor function scale; C, the communication scale; R, the arousal scale; FA, fractional anisotropy; ADC, apparent diffusion coefficient; t1, 1.5 months after injury; t2, 7.5 months after injury; and t3, 17.5 months after injury. The maximum possible score on each subscale is denoted by the thick black lines

The patient presented with a diagnosis of UWS [19], which was confirmed by experienced raters. The CRS-R scores were based on the best response in the repeated behavioral assessments (5 repeated assessments in the separate days of a week). In our first evaluation (1.5 months after injury), there was evidence of behavioral responsiveness (Fig. 2a) with a CRS-R score of 4 (auditory function = 0; visual function = 0; motor function = 2; verbal function = 0; communication ability = 0; arousal level = 2). Notably, while the patient showed no signs of awareness, this was consistent with the definition of UWS. The CRS-R score had increased to 12 (2/3/2/2/0/3) after 6 months (7.5 months after injury) and re-evaluation suggested evidence of changes in the patient’s state of consciousness, such as sound source localization, visual tracking, and flexion of lower limbs (Fig. 2b). These improvements allowed the patient’s diagnosis to change to MCS [20]. The third assessment at 17.5 months after injury (1.5 years after first evaluation) provided a CRS-R score of 14 (2/4/2/2/1/3), which indicated the improvement of cognitive and behavioral abilities with object localization, localization to noxious stimulation, and non-functional communication (Fig. 2c).

### Analyses using routine DTI evaluation parameters

We calculated the average FA and ADC values for the patient at three-time points during the follow-up period and depicted the trend of changes in the brain microstructure (Fig. 2d and e). (The changes in AD and RD were presented in Figure S2). The declining FA and increasing ADC values represented further aggravation of brain microstructural damage.

### Analyses using diffusion connectometry

**Fig. 3:**
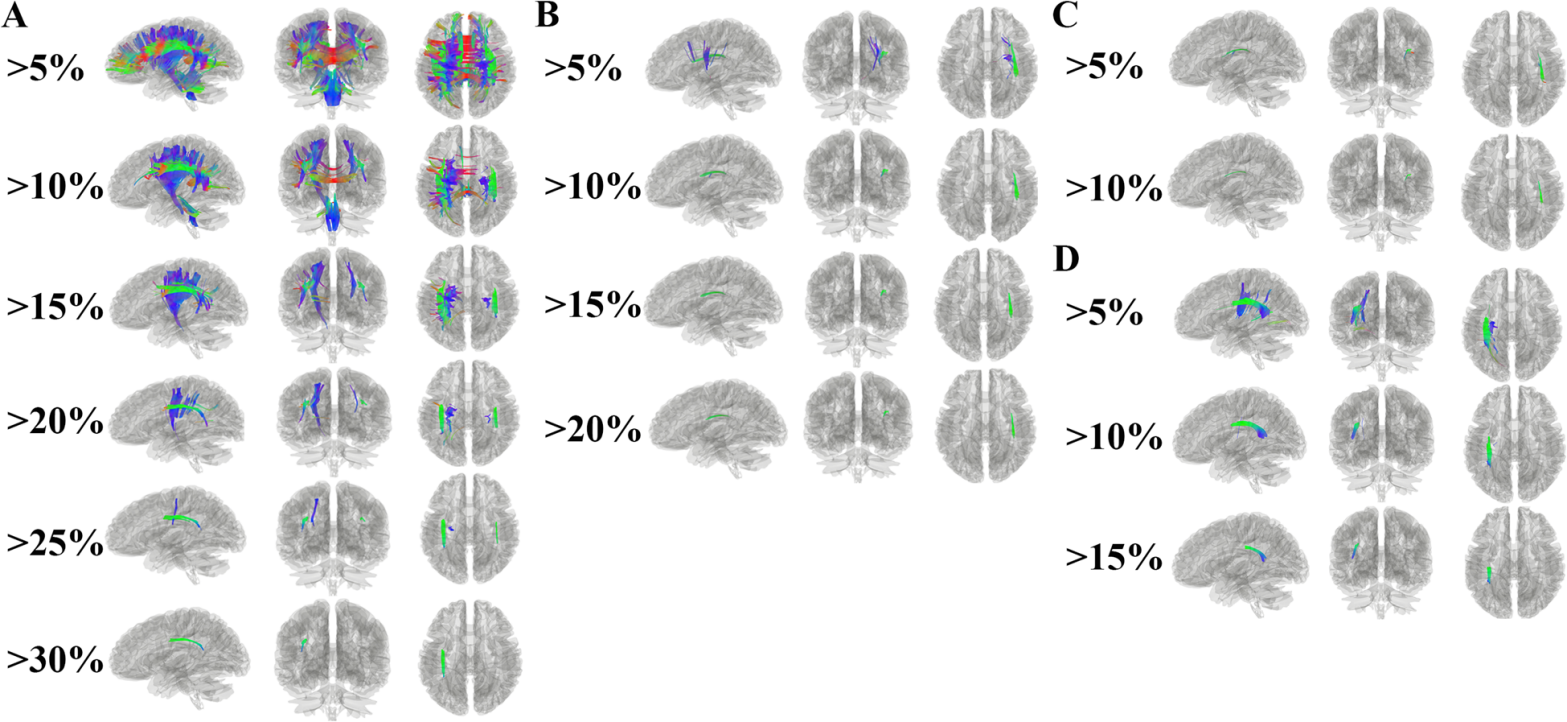
Fiber density varying over time. **a**: Tracts with significantly reduced density during t1-t2. Left SLF and Left AF were the tracts with the most decreased fiber densities. **b**: Tracts with increased density during t1-t2. Right SLF and Right AF were the tracts with the most increased fiber densities. **c**: Tracts with reduced density during t2-t3. Right SLF and Right AF were the tracts with the most decreased fiber densities. **d**: Tracts with significantly increased density during t2-t3. Left SLF and Left AF were the tracts with the most increased fiber densities. Red: left–right, green: anterior–posterior, and blue: superior–inferior. t1, 1.5 months after injury; t2, 7.5 months after injury; and t3, 17.5 months after injury

The connectometry analysis revealed changes of the structure and density of the white matter tracts at three time points during follow-up (Fig. 3). The main period for the decrease of fiber density was t1-t2, and the main area was located in the left hemisphere, particularly arcuate fasciculus (AF) and superior longitudinal fasciculus (SLF) (Fig. 3a). The main period for the increase of fiber density was t2-t3, and the tracts increased mostly in left AF, left SLF, and left optic radiation (Fig. 3d). Increased fiber density during t1-t2 was mainly located in the right hemisphere of the brain, of which AF and SLF increased obviously (Fig. 3c). During t2-t3, the obvious decrease in fiber density was right AF and right SLF (Fig. 3b). The FA, ADC, RD and AD values on SLF/AF were presented in Figure S1 and Figure S3.

### Analyses using a graph theory analysis

**Fig. 4:**
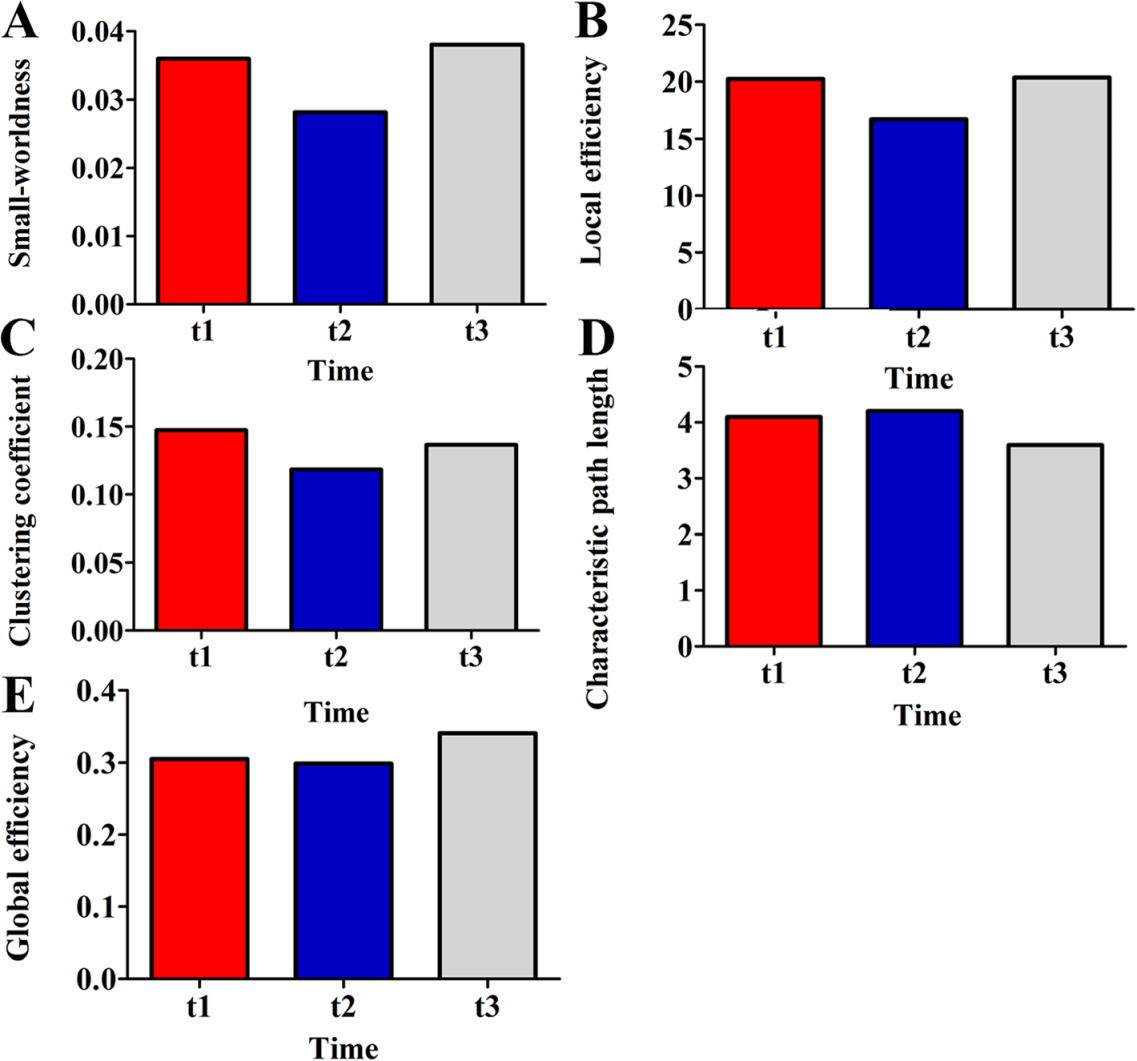
The global topologic network parameters at different time points. The horizontal scale indicated different follow-up times, and the vertical scale indicates the value of the topology metrics. t1, 1.5 months after injury; t2, 7.5 months after injury; and t3, 17.5 months after injury

The global graph metrics were calculated to investigate possible differences in the overall network topology during the longitudinal follow-up process [21]. During t1-t2, the small world-ness, the local efficiency, clustering coefficient and global efficiency showed a decreasing trend but the characteristic path length showed an increasing trend. During t2-t3, the small world-ness, the local efficiency, clustering coefficient and global efficiency turn to increased but the characteristic path length turned to decreased. The changes of above graph theory metrics are presented in Fig. 4.

## Discussion and conclusions

The primary purpose of the present study was to explore changes in brain microstructure and the topologic properties of the brain network, combined with behavioral assessments, to comprehensively evaluate the disease progression for 1.5 years over a DOC patient.

After the behavioral assessments, we believed that the condition of the DOC patient was improving as the visual, auditory, and linguistic functions showed apparent improvements, which was consistent with previously reported studies [22, 23]. We found that the visual function demonstrated the most obvious improvements. When we combined these characteristics with T1-weighted images, the enlarged ventricles and obvious oedema and/or gliosis near the frontal horn of the lateral ventricles indicated a poor prognosis. As for the diffusion metrics, previous studies have shown that low FA and high ADC indicated a poorer prognosis [24–26]. Notably, the results were controversial. Therefore, further whole-brain analysis was necessary.

We used the connectometry method to further analyze the changes of whole-brain fibers. During t1-t2, the decrease in fiber density was prominent in the left projection, association and commissural fibers, suggesting that the injury was more severe in the left hemisphere. Meanwhile, increased fiber density was also found in the right SLF, which may indicate a compensatory response (the progenitor cells of oligodendrocytes, under certain conditions, can proliferate, differentiate and migrate to damaged areas for repair [27]). During t2-t3, the increased fiber density mainly dominated in the left hemisphere, which may result from new sprout axons [28] and the remission of edema at the initial traumatic area. Besides, tracts with decreased fiber density were less involved in the right hemisphere compared with the t1-t2 period, and it may be attributed to the secondary damage after the primary insult [29–31].

The SLF/AF in the bilateral hemispheres accounted for the largest percentage of fiber density alterations. The SLF is a large bundle of associated tracts in the white matter and is thought to serve as a conduit for the neural system promoting visual awareness, the maintenance of attention, and engagement in the environment [32, 33]. These associations are largely due to its link to the angular gyrus and the parieto-occipital areas, as well as the posterior part of the dorsolateral and mid-dorsolateral prefrontal cortex [34, 35]. The AF is the fourth component of the SLF, connecting various functional areas within the temporal, parietal, and frontal lobes [36, 37]. It is important for the spatial properties of auditory stimuli and auditory-related processing. Historically, the SLF/AF system has been the predominant white matter in language studies for more than 150 years. The SLF/AF is a connection between receptive and expressive language centers [38, 39], and is involved in sensorimotor processes that support speech production and speech perception [40]. Given this theoretical basis, the large improvement in the visual, auditory, and verbal functions represented by the CRS-R scale may be associated with the alteration of SLF/AF. As for the visual function, its recovery might be related to the microstructural changes of optic radiation together with the SLF/AF [41].

We used graph theory analyses to systematically study the pathologic white matter network reorganization in one patient, and five graph theory metrics were described. The reduced small world-ness attributes from t1-t2 represent the broken of optimal communication efficiency. From the perspective of information separation, the weaken in clustering coefficients and local efficiencies represent a decrease in the modularity of the brain. From the perspective of information integration, the increase of the characteristic path length, and the reduction in the global efficiency represent the shrinking transmission capability of the brain. From t2 to t3, the changes of aforementioned metrics represented the improvement of whole brain network integrity and information processing ability.

The conclusions drawn from graph theory and connectometry studies differed from that of the CRS-R. We assumed that this difference was due to coexisting damage and repair of the microstructural and topologic properties in the whole brain of the patient. From t1-t2, the damaged portion of the brain was too severe in a relative sense and cover the repaired portion of the fiber density. Improved regions can be reflected in the patient’s clinical manifestations; however, during the t2-t3, both the trend of improved fiber density and topological properties suggest the possibility of brain developing in a better direction.

Through this comprehensive assessment, we speculate that 1 year might not be the optimal to announce the eventual extent of recovery of brain. In our present study, although the DOC course had lasted more than 1 year, we were still optimistic that the brain function still had the possibility to develop towards a better state. This case report could provide a reference for the clinical definition of treatment time windows for all DOC patients. Additionally, the preliminary discovery of the SLF and AF involvement was necessary to advance the understanding of the underlying mechanisms associated with DOC.

Notably, our study had potential limitations. First of all, after a follow-up of 7.5 months, the patient was discharged from the hospital. Due to the patient’s discomfort and inconvenience for long-distance transportation, the detection time point was not very rigorous. Although we followed up for 1.5 years, this was only a single patient study, and enrolling more patients is preferable to confirm the role of the SLF/AF further. Additionally, changes to the intracranial microstructure and topologic properties of DOC patients could be different due to the different disease types. DOC caused by ischemia with decreased oxygen levels and hemorrhage should also be observed for a longer time to have more insights of the spontaneous changes in the course of DOC and to find more convincing evidence to define a more appropriate recovery time window.

In conclusion, the SLF/AF was the fiber bundle exhibiting the greatest fiber density alterations, suggesting that the SLF/AF could be important in the progression of DOC. The changes to the network topologic properties were consistent with trends in fiber density changes, potentially reflecting the neural fundamentals of the recovery course. Comprehensive analysis results could detect subtle longitudinal alterations and may be a beneficial tool for monitoring patients with DOC in the chronic period.

## Supplementary Information

**Additional file 1:** **Table S1.** Mathematical definitions and descriptions of the network metrics used in this study. **Figure S1**. The FA (A) and ADC (B) values at three timepoints (t1:1.5 M, t2:7.5 M t3:17.5 M). **Figure S2**. The average AD and RD values of whole brain at three timepoints (t1:1.5 M, t2:7.5 M t3:17.5 M). **Figure S3**. The AD (A) and RD (B) values at three timepoints (t1:1.5 M, t2:7.5 M t3:17.5 M).

## Data Availability

The datasets generated and/or analyzed during the current study are not publicly available due to institutional restrictions but may be available upon request to corresponding authors.

## Abbreviations

DOC: Disorders of consciousness
CRS-R: Coma Recovery Scale-Revised
MRI: Magnetic resonance imaging
DTI: Diffusion tensor imaging
T1WI: T_1_-weighted imaging
TE: Echo time
TR: Repetition time GRAPPA Generalized auto-calibrating partially parallel acquisitions
TA: Acquisition time
ADC: Apparent diffusion coefficient
FA: Fractional anisotropy
UWS: Unresponsive wakefulness syndrome
MCS: Minimally conscious state
SLF: Superior longitudinal fasciculus
AF: Arcuate fasciculus
