# Supplementary material for: Chronic disorders of consciousness: a case report with longitudinal evaluation of disease progression using 7 T magnetic resonance imaging
Source: BMC Neurol. 2020 Oct 29;20:396. doi: 10.1186/s12883-020-01973-0 (PMC7594973; doi:10.1186/s12883-020-01973-0)
Supplement: Supplementary file 1 — Additional file 1: Table S1. Mathematical definitions and descriptions of the network metrics used in this study. Figure S1. The FA (A) and ADC (B) values at three timepoints (t1:1.5 M, t2:7.5 M t3:17.5 M). Figure S2. The average AD and RD values of whole brain at three timepoints (t1:1.5 M, t2:7.5 M t3:17.5 M). Figure S3. The AD (A) and RD (B) values at three timepoints (t1:1.5 M, t2:7.5 M t3:17.5 M). [file 12883_2020_1973_MOESM1_ESM.pdf]

Table S1. Mathematical definitions and descriptions of the network metrics used in this study

Network metrics	Definition	Description
Density	$\rho = \frac{1}{N(N-1)} \sum_{i,j=1}^{N} A_{i,j}$	Averaging all the entries of the adjacency matrix gives the density (ρ) of the network as the percentage of significant edges that are present out of the total number of edges possible (Freeman, 1977, 1979).
Clustering coefficient	$C_p(G) = \frac{1}{N} \sum_{i=1}^{N} \frac{\sum_{j,k \in N} \left(\overline{w_{ij} w_{jk} w_{ki}}\right)^{1/3}}{k_i (k_i - 1)}$	k_i is the degree of node *i*. The clustering coefficient reflects the cliquishness of a typical neighborhood in a network and measures the segregation between brain regions (Onnela et al., 2005).
Network characteristic path length	$L_p(G) = \frac{1}{N(N-1)} \sum_{i=1}^{N} \sum_{j=1, j \neq i}^{N} L_{ij}$	Shortest path length L_{ij} between node *i* and node *j* ($i \neq j$) is the reciprocal of edge weight, $1/w_{ij}$. A shorter path length implies a stronger potential for the integration of information flow between brain regions (Watts and Strogatz, 1998).
Small world-ness	$S = \frac{C / C_{rand}}{L / L_{rand}}$	where C and C_{rand} are the clustering coefficients, and L and L_{rand} are the characteristic path lengths of the respective tested network and a random network. Small-world networks often have $S \gg 1$ (Humphries and Gurney, 2008).
Global efficiency	$E_{glob}(G) = \frac{1}{N(N-1)} \sum_{i=1}^{N} \sum_{j=1, j \neq i}^{N} \frac{1}{L_{ij}}$	E_{glob} is computed on disconnected networks. Paths between disconnected nodes are defined to have infinite length and correspond to zero efficiency (Latora and Marchiori, 2001).
Local efficiency	$E_{loc}(G) = \frac{1}{N} \sum_{i=1}^{N} E_{glob}(G_i)$	G_i denotes the subgraph composed of the nearest neighbors of node *i*, and $E_{glob}(G_i)$ is the global efficiency of the subgraph G_i. The local efficiency represents how much the network is fault tolerant and how efficient the communication is between the first neighbors of node *i* when node *i* is removed (Latora and Marchiori, 2001).

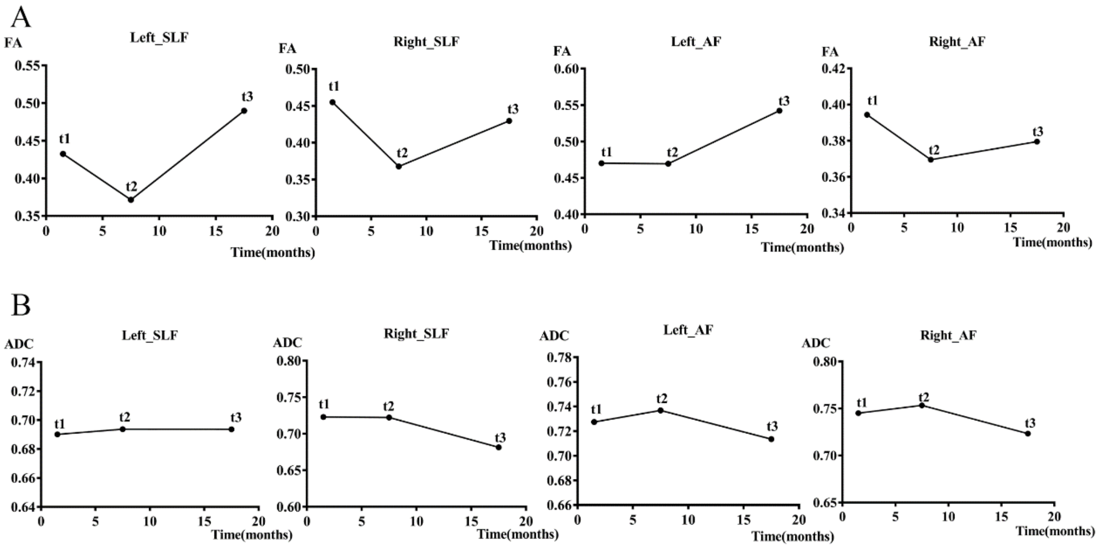

Fig.S1. The FA (A) and ADC (B) values at three timepoints (t1:1.5M, t2:7.5M t3:17.5M).

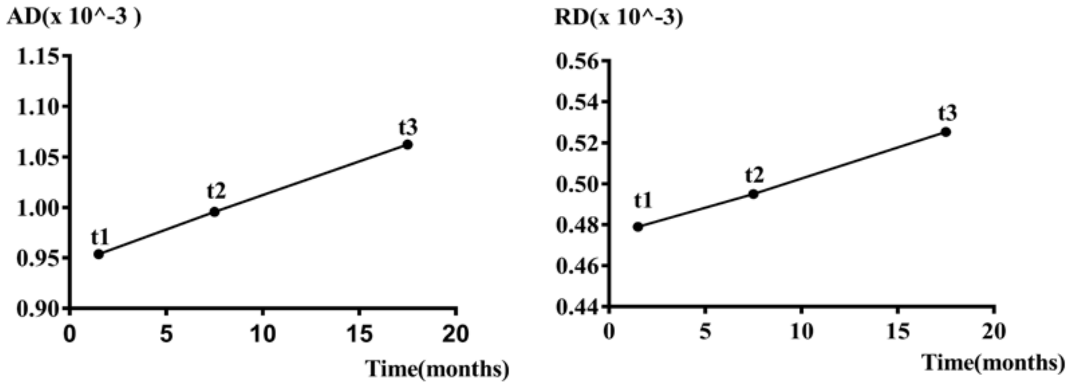

Fig.S2. The average AD and RD values of whole brain at three timepoints (t1:1.5M, t2:7.5M t3:17.5M).

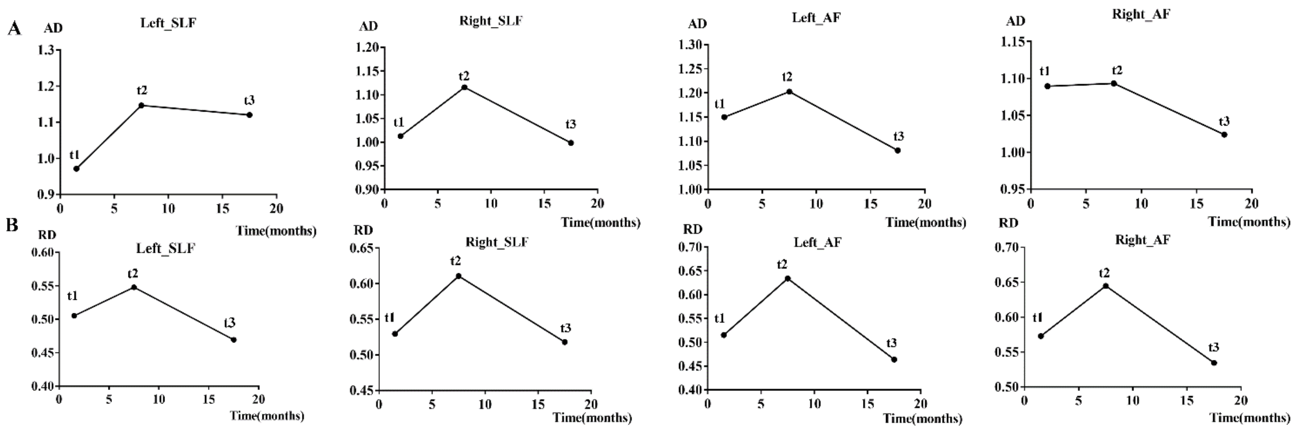

Fig.S3. The AD (A) and RD (B) values at three timepoints (t1:1.5M, t2:7.5M t3:17.5M).
